# Targeting the secretory autophagy–extracellular vesicle interface: fate decisions and translational opportunities

**DOI:** 10.3389/fphar.2026.1958455

**Published:** 2026-09-29

**Authors:** Linlin Deng, Linwei Li, Zhijun Huang, Changfeng Huang

**Affiliations:** Department of Stomatology, The People’s Hospital of Yubei District of Chongqing, Chongqing, China

**Keywords:** autophagosome fate, extracellular vesicles, lysosomal function, membrane trafficking, pharmacological modulation, secretory autophagy, therapeutic targets, translational pharmacology

## Abstract

Autophagy preserves cellular homeostasis through lysosomal degradation, but its machinery also participates in extracellular release. This creates a pharmacological problem: changing autophagy or extracellular vesicle (EV) output can alter intracellular disposal, inflammatory signaling and communication between cells in different directions. This Review distinguishes lysosomal degradation, direct secretory autophagy and EV-associated release, with particular attention to cargo selection, tethering, fusion and the limits of route assignment. We define secretory autophagy during lysosome inhibition as a stress-dependent response and distinguish it from regulated secretion in physiological settings. Disease comparisons across cancer, neurodegeneration, inflammation and infection show why the source cell, cargo and recipient response matter for intervention. We evaluate three strategies: restoring degradation, suppressing harmful secretion and preserving or exploiting EV-linked communication. For representative interventions, we separate pharmacological tools from genetic candidate targets and pathway-level effects, and compare functional evidence, risks and translational maturity. Dynamic imaging, single-EV profiling and orthogonal perturbations offer complementary approaches to test route-specific effects. Current evidence supports preclinical opportunities, but does not establish clinical efficacy through a specifically validated secretory-autophagy–EV mechanism.

## 1 Introduction

Autophagy is commonly defined as a lysosome-dependent degradative pathway in which proteins, aggregates, and organelles are sequestered within double-membrane autophagosomes and delivered for breakdown (Deretic et al., 2012; Ponpuak et al., 2015; New and Thomas, 2019). Although degradation remains its canonical function, this definition does not account for every extracellular outcome involving autophagy machinery. Autophagy-related (ATG) modules, microtubule-associated protein 1 light chain 3 (LC3) conjugation systems, and associated trafficking factors can also mediate non-degradative release of soluble and membrane-associated cargo (Kimura et al., 2017; Li et al., 2024; Noh et al., 2022). The relevant mechanistic issue is therefore how an autophagy-related membrane is committed to degradation or extracellular release.

Extracellular vesicles (EVs) are directly relevant to this question. These heterogeneous membrane-enclosed particles mediate intercellular communication, stress responses, and extracellular disposal (Buratta et al., 2020; Jeppesen et al., 2019). The endosome-derived EV subset intersects with autophagy through endolysosomal maturation, acidification, multivesicular body (MVB) trafficking and plasma membrane fusion (Ostrowski et al., 2010; Larios et al., 2020; Choezom and Gross, 2022; Matsui et al., 2023). These mechanisms should not be generalized to every EV population. Mechanistic links include the detection of LC3-II in EV fractions, changes in EV loading or release after ATG perturbation, and ATG12-ATG3 regulation of late-endosome function through the endosomal sorting complexes required for transport (ESCRT) accessory protein apoptosis-linked gene 2-interacting protein X (ALIX; also known as PDCD6IP). In addition, lysosome-associated membrane protein 2A (LAMP2A) and the chaperone heat shock 70-kDa protein 8 (HSC70) mediate the loading of KFERQ-like cargo into a subset of exosomes (Leidal et al., 2020; Leidal and Debnath, 2021; Gardner et al., 2023; Xu et al., 2022; Murrow et al., 2015; Ferreira et al., 2022; Colombo et al., 2013). These observations warrant joint analysis of autophagy-linked extracellular output and EV biology, provided that their distinct routes are not treated as interchangeable.

Previous reviews have addressed LC3-dependent EV loading and secretion, extracellular output during lysosomal inhibition, unconventional secretion of leaderless proteins, and examples from cancer and neurodegeneration (Li et al., 2024; Noh et al., 2022; Buratta et al., 2020; Leidal and Debnath, 2021; Zubkova et al., 2024). A persistent problem is route assignment. Direct secretory autophagy, EV-associated cargo loading, rerouting after lysosomal failure, and lysosomal exocytosis can yield similar extracellular readouts (Xu et al., 2022; Solvik et al., 2022; Shelke et al., 2023; Mao et al., 2025), although they are mechanistically distinct. We therefore use autophagosomal fate as an organizing principle, without implying that all extracellular outputs arise through a common pathway.

We focus on extracellular outputs that involve autophagy machinery and intersect with EV biology. The pharmacological question is whether an intervention changes a defined cargo route in a way that benefits the donor cell, the recipient cell or the affected tissue. Contrasting effects of lysosomal inhibition, tumor-associated fate redirection and treatment-induced high mobility group box 1 (HMGB1) secretion show why a change in total EV output is insufficient to answer this question (Solvik et al., 2022; Li et al., 2022; Wei et al., 2024). We first distinguish the routes and their physiological and disease contexts, then compare restoration of degradation, suppression of harmful secretion and preservation or exploitation of EV-linked communication. We assess representative pharmacological and genetic evidence, while separating preclinical mechanism from clinical validation.

Our pharmacological analysis compares interventions, target or pathway evidence, disease-relevant outcomes, and constraints on selectivity, exposure and toxicity. It examines how drugs alter cargo routing and how those changes affect donor and recipient cells. Endogenous cargo and metabolite trafficking are considered separately from drug disposition, because they do not establish drug metabolism or transport. Our discussion of EV coupling focuses on endogenous cargo transfer and treatment-associated changes in extracellular communication.

## 2 The autophagy-EV interface: shared machinery and divergent output routes

Autophagy proteins participate in several membrane-trafficking routes. Cargo-containing compartments may undergo lysosomal degradation, fuse with the plasma membrane or contribute to EV-associated release, but not every route requires autophagosome formation (Leidal et al., 2020; Solvik et al., 2022). These outcomes must be distinguished before their regulators are inferred, because a similar extracellular readout does not establish a shared mechanism.

Accordingly, we use operational evidence, rather than terminology alone, to assign each route.

Direct secretory autophagy refers to export through fusion of an autophagy-related compartment with the plasma membrane, ideally supported by evidence for the terminal fusion machinery and for non-vesicular release of the cargo.

EV-associated autophagy-linked secretion refers to membrane-enclosed extracellular release in which autophagy machinery contributes to cargo loading, compartment maturation, or vesicle release.

Secretory autophagy during lysosome inhibition (SALI) refers to a lysosomal inhibition state that selectively redirects autophagy-linked cargo outward and should not be used as a blanket term for all extracellular autophagic output.

Lysosomal exocytosis refers to plasma membrane fusion of lysosomes and should be distinguished from both direct secretory autophagy and EV-associated release even when the same stress condition increases all three outputs.

### 2.1 Shared upstream machinery and route-specific requirements

Macroautophagy begins with an initiation complex containing the kinase UNC-51-like kinase 1 (ULK1), ATG13, ATG101 and focal adhesion kinase family-interacting protein of 200 kDa (FIP200). The assembled complex coordinates early membrane formation (Deretic et al., 2012; Leidal and Debnath, 2021). Downstream membrane expansion and lipidation of LC3 or GABA type A receptor-associated protein (GABARAP) involve the ATG5-ATG12-ATG16L1 conjugation system (Deretic et al., 2012; Li et al., 2024; Leidal and Debnath, 2021). Of relevance here is the recurrence of many of these upstream modules in routes that do not end in degradation (Noh et al., 2022; Zubkova et al., 2024). LC3 conjugation is particularly important, since it participates not only in autophagosome maturation but also in route-specific cargo loading into extracellular vesicle-associated outputs (Leidal et al., 2020; Gardner et al., 2023; Leidal and Debnath, 2020). Overlapping ATG requirements can explain why degradative and secretory phenotypes respond to some of the same perturbations, without implying a common membrane origin for every output (Leidal et al., 2020; Xu et al., 2022; Wang et al., 2023). This overlap also explains why non-canonical ATG functions, such as ATG12-ATG3 control of ALIX-dependent late endosome behavior, can influence extracellular vesicle output in ways that are mechanistically adjacent to, but distinct from, canonical macroautophagy (Murrow et al., 2015).

The relevant distinction is between shared machinery and shared organelle biogenesis. In LC3-dependent EV loading and secretion (LDELS), LC3 conjugation supports EV cargo loading, whereas loss of the early autophagy factors ATG14 or FIP200 did not suppress the tested LC3-associated EV outputs (Leidal et al., 2020). By contrast, SALI cargo-receptor export required factors involved in phagophore formation, LC3 conjugation and autophagosome completion (Solvik et al., 2022). A recent study further separated EV quantity from autophagic cargo export: ATG9A or ATG2A/B loss increased CD63-enriched small-EV secretion but prevented loading of LC3 and canonical autophagy receptors under lysosomal inhibition (Singh et al., 2026). ATG2 and ATG9 therefore influence membrane supply and cargo routing; their loss does not imply that all EV biogenesis is inhibited.

### 2.2 Route I: autophagosome-lysosome fusion and degradative output

The degradative route provides the reference for assessing extracellular diversion. Its components have distinct roles: RAB7 is a small GTPase that recruits trafficking effectors, tethering factors bring compartments together, and soluble N-ethylmaleimide-sensitive factor attachment protein receptor (SNARE) proteins assemble into complexes that execute membrane fusion (Wang et al., 2016; Jian et al., 2024; van der Beek et al., 2024). Syntaxin 17 (STX17) is a Qa-SNARE on mature autophagosomes; synaptosome-associated proteins SNAP29 and SNAP47 provide Qb/Qc SNARE motifs; and vesicle-associated membrane proteins VAMP7 or VAMP8 supply the R-SNARE partner. These names denote individual proteins, whereas STX17–SNAP47–VAMP7/VAMP8 denotes a fusion complex with alternative VAMP partners. SNAP47-containing complexes supported both selective autophagy under non-starvation conditions and starvation-induced autophagy in the tested systems; SNAP29-containing complexes cannot be assumed to substitute in every setting (Jian et al., 2024).

Fusion competence is regulated before the final membrane-merger event. Phosphatidylinositol 4-phosphate (PI4P) accumulation contributes to STX17 recruitment to mature autophagosomes (Shinoda et al., 2024). YKT6, another SNARE protein, was found to form a priming complex with STX17 and SNAP29 that facilitates subsequent STX17–SNAP29–VAMP8-mediated fusion, illustrating cooperation rather than simple substitution (Zheng et al., 2024). Sec1 family domain-containing protein 1 (SCFD1) is a SNARE-assembly regulator whose acetylation inhibited assembly of this fusion complex in the tested cells (Huang et al., 2023). These studies identify regulation of recruitment, priming and assembly as distinguishable stages; they do not establish that every regulator directly switches cargo into secretion.

HOPS (homotypic fusion and vacuole protein sorting) is a six-subunit tethering complex comprising VPS11, VPS16, VPS18, VPS33A, VPS39 and VPS41, rather than a single fusion protein (van der Beek et al., 2024). The Vici syndrome protein EPG5 is a distinct RAB7 effector and tethering factor that stabilizes the autophagosomal SNARE assembly and contributes to fusion specificity (Wang et al., 2016). Thus, a tether, a small GTPase and a SNARE cannot be treated as interchangeable members of one protein family. Their coordination establishes a competent fusion site before lysosomal degradation can occur.

### 2.3 Route II: secretory autophagy via plasma membrane fusion

In direct secretory autophagy, autophagic cargo is exported through fusion of autophagosomal or amphisomal structures with the plasma membrane rather than with lysosomes (Ponpuak et al., 2015; New and Thomas, 2019; Noh et al., 2022). This route is best established for leaderless proteins that bypass conventional endoplasmic reticulum (ER)-Golgi secretion. Early work in *Dictyostelium* showed that Golgi reassembly stacking protein (GRASP) was required for unconventional secretion of acyl-CoA-binding protein AcbA during development; that study left the membrane route unresolved (Kinseth et al., 2007).

Interleukin-1β (IL-1β) is among the best-characterized mammalian examples. Autophagy machinery contributes to its cargo capture and export through an unconventional pathway rather than degradative turnover (Dupont et al., 2011; Zhang et al., 2015; Claude-Taupin et al., 2018). Transmembrane emp24 domain-containing protein 10 (TMED10) was subsequently identified as a channel-like factor that translocates leaderless cargo into endoplasmic reticulum–Golgi intermediate compartment (ERGIC)-related vesicular intermediates (Zhang et al., 2020). This finding helped explain how soluble cytosolic cargo enters a membrane-bound secretory route. In the IL-1β pathway, tripartite motif-containing protein 16 (TRIM16) acts as a cargo receptor and interacts with the R-SNARE SEC22B to recruit cargo to LC3-positive membranes. Secretion involves SEC22B together with syntaxins 3 or 4 and SNAP23 or SNAP29, whereas STX17 depletion did not inhibit this output (Kimura et al., 2017). This is evidence for distinct cargo-selection and terminal-fusion requirements, not a universal replacement of degradative SNAREs in every secretory pathway.

Direct secretory autophagy is conceptually distinct from extracellular vesicle loading. Its hallmark is not vesicular encapsulation into exosome-like particles, but plasma membrane fusion of autophagy-related compartments carrying soluble or unconventionally secreted cargo. HMGB1 follows the same general logic, though its secretion involves additional steps including stress-responsive nuclear export and cooperation with endolysosomal trafficking (Li et al., 2022; Kim et al., 2021). Other cargoes broaden this route without collapsing it into a single mechanism. Misfolded cystic fibrosis transmembrane conductance regulator (CFTR) can use a GRASP-dependent unconventional trafficking route, and VAMP7-linked SNARE machinery has been discussed more broadly in unconventional secretion (Vats and Galli, 2022; Gee et al., 2011). The amphisome is an important intermediate, because fusion between autophagosomes and late endosomal compartments can generate hybrid organelles with either degradative or secretory potential (Buratta et al., 2020; Ganesan and Cai, 2021; Fader et al., 2008; Visnovitz et al., 2025). Route II is therefore defined less by one unique compartment than by its terminal decision to fuse with the cell surface (Kimura et al., 2017).

### 2.4 Route III: EV-associated secretory outputs

A third route uses autophagy-related machinery to shape extracellular vesicle-associated output. The most clearly defined example is LDELS, in which LC3 conjugation promotes the incorporation of selected cargoes into extracellular vesicles (Leidal et al., 2020; Leidal and Debnath, 2021; Leidal and Debnath, 2020). LC3 binds selected RNA-binding cargoes, including heterogeneous nuclear ribonucleoprotein K (HNRNPK) and scaffold-attachment factor B (SAFB). Neutral sphingomyelinase 2 (nSMase2) and its regulator FAN (factor associated with nSMase2 activity) support this endosomal loading process (Leidal et al., 2020). RAB27A supports vesicle release. The route also accommodates transmembrane cargo, including the transferrin receptor (TFRC), and is therefore not limited to soluble ribonucleoprotein-associated cargo (Gardner et al., 2023). EV-associated output is mechanistically heterogeneous. ATG12-ATG3 links autophagy proteins to ALIX-dependent late endosome function and exosome biogenesis, whereas LAMP2A-HSC70 loads KFERQ-like cargo into a subset of exosomes. ALIX-ESCRT and plasma membrane SNARE modules further regulate tetraspanin sorting and multivesicular body fusion with the cell surface (Larios et al., 2020; Matsui et al., 2023; Murrow et al., 2015; Ferreira et al., 2022; Colombo et al., 2013; Verweij et al., 2018).

Shared regulators also operate outside a demonstrated secretory-autophagosome route. Rubicon, the RUN domain Beclin-1-interacting and cysteine-rich domain-containing protein, inhibits autophagy but also supports exosome biogenesis. It recruits WIPI2d, an isoform of WD repeat domain phosphoinositide-interacting protein 2, to endosomes. WIPI2d interacts with ESCRT machinery involved in intraluminal vesicle formation. This exosome function was independent of autophagy in the tested systems (Yanagawa et al., 2024). ESCRT comprises membrane-remodeling assemblies rather than one protein. Similarly, a SNARE screen found that SNAP29 depletion reduced exosome-associated proteins without reducing extracellular LC3B or p62 in the tested cells (Hessvik et al., 2023). These findings reinforce the need to identify the cargo and vesicle population affected by a shared trafficking factor.

Route III differs from direct secretory autophagy in that cargo is released within membrane-enclosed extracellular vesicles rather than by simple fusion-mediated discharge. It also differs from lysosomal stress-induced extracellular output, although stress can reshape EV composition. Chloroquine treatment, for instance, promotes secretion of autophagy-related proteins and inclusion of ATG8 family proteins in distinct extracellular vesicle populations, demonstrating the influence of endolysosomal status on EV-associated output (Xu et al., 2022; Solvik et al., 2022). nSMase2 activity, phosphoinositide conversion, and endosomal acidification likewise modulate exosome secretion, consistent with evidence that EV production depends on endosomal fate decisions that intersect with, but are not reducible to, canonical autophagy (Choezom and Gross, 2022; Matsui et al., 2023; Liu et al., 2023; Verweij et al., 2022).

### 2.5 Cargo profiles as clues to route utilization

Route utilization leaves characteristic, but non-exclusive, cargo signatures. Direct secretory autophagy is commonly associated with leaderless immune-related proteins such as IL-1β and HMGB1 (Kimura et al., 2017; Dupont et al., 2011; Kim et al., 2021). LDELS-type EV secretion is marked by selective export of RNA-binding proteins, small non-coding RNAs, and certain transmembrane proteins such as TFRC (Leidal et al., 2020; Gardner et al., 2023; Leidal and Debnath, 2020). Lysosomal stress-associated extracellular output, by contrast, is enriched for cargo receptors, including sequestosome 1 (SQSTM1/p62) and neighbor of BRCA1 gene (NBR1), together with LC3-family proteins (Xu et al., 2022; Solvik et al., 2022). These signatures are informative but do not define a route (Zubkova et al., 2024). Because some autophagy receptors and soluble cytosolic cargoes can also enter late endosomal vesicles through endosomal microautophagy, especially under acute starvation, cargo identity alone does not prove route identity (Sahu et al., 2011; Mejlvang et al., 2018). Figure 1 summarizes the major routes and biological contexts, whereas Figure 2 integrates representative cargo-selection, tethering and fusion modules with modification-state control and context-dependent outcomes. Table 1 defines the route categories, and Table 2 summarizes cargo classes and the evidence needed to distinguish their routes. How cells select among these routes remains unresolved.

**FIGURE 1:**
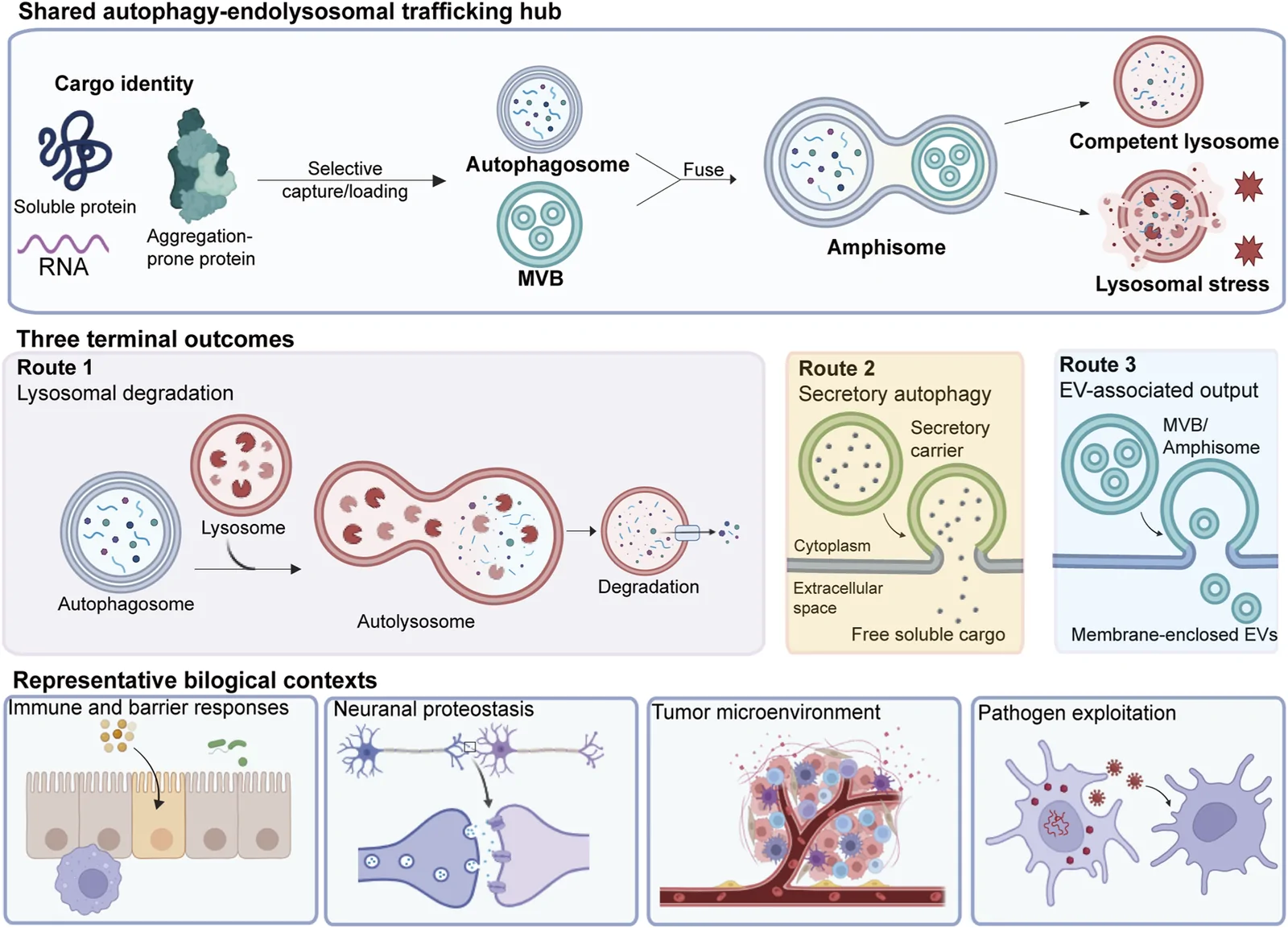
Fate decisions at the shared autophagy–endolysosomal trafficking hub. Soluble proteins, RNA, and aggregation-prone proteins can enter autophagosome- or multivesicular body-associated compartments and converge at amphisomes. Lysosomal competence favors degradative delivery, whereas lysosomal stress can bias extracellular rerouting. The schematic distinguishes three terminal outcomes: lysosomal degradation, direct secretory autophagy with release of free soluble cargo, and extracellular vesicle-associated output with membrane-enclosed cargo. Representative biological contexts include immune and barrier responses, neuronal proteostasis, the tumor microenvironment, and pathogen exploitation.

**FIGURE 2:**
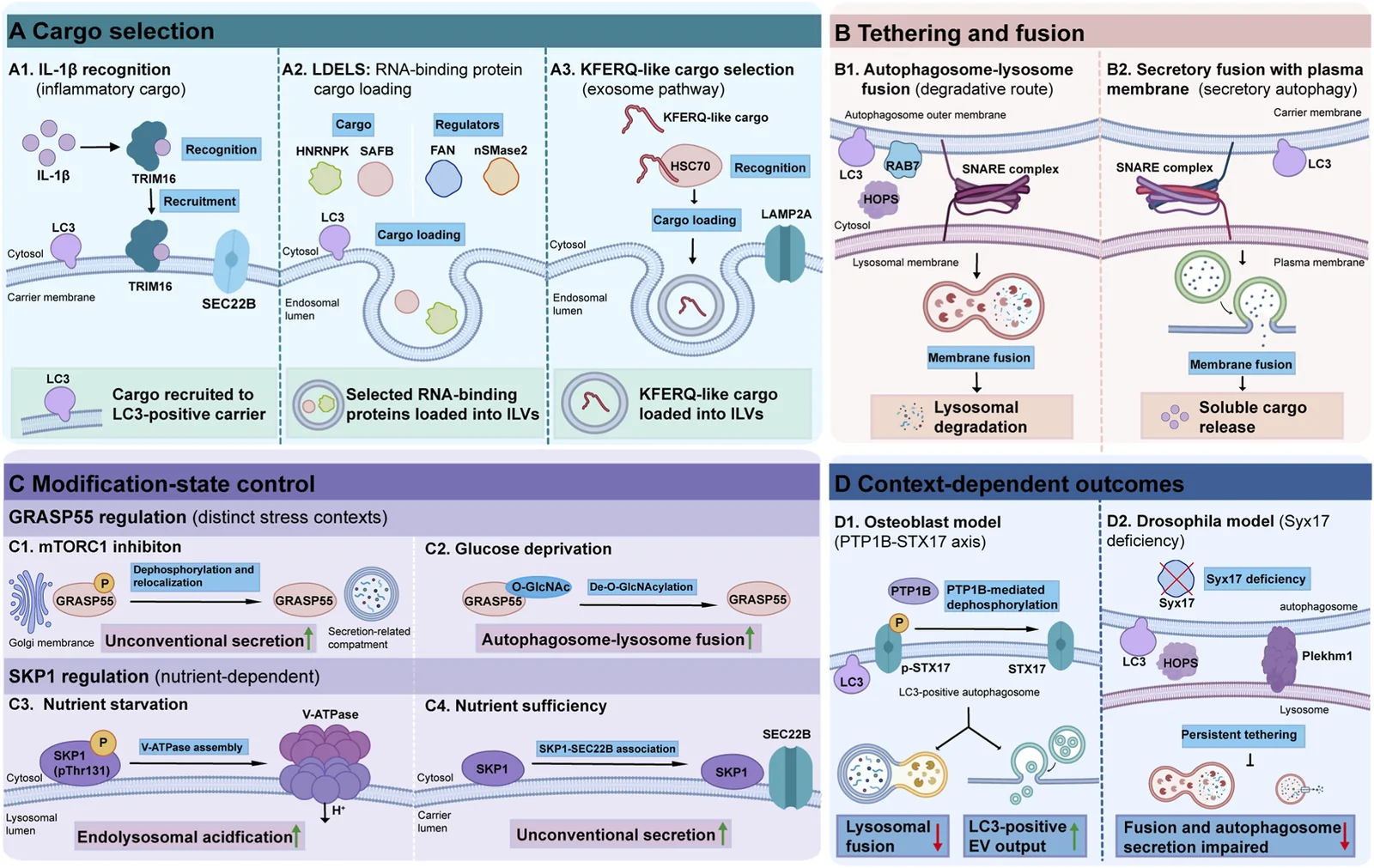
Selected molecular mechanisms at the secretory autophagy–extracellular vesicle interface. **(A)** Cargo selection. **(A1)**, TRIM16 recognizes IL-1β and associates with SEC22B to recruit cargo to LC3-positive carrier membranes (Kimura et al., 2017). **(A2)** LC3-dependent extracellular vesicle loading and secretion (LDELS) supports endosomal loading of RNA-binding proteins, including HNRNPK and SAFB, with contributions from FAN and nSMase2 (Leidal et al., 2020). **(A3)** HSC70 and LAMP2A support loading of KFERQ-like motif-containing cargo into intraluminal vesicles (ILVs) through a distinct endosomal mechanism (Ferreira et al., 2022). These modules do not imply a common obligatory autophagosome or amphisome intermediate. **(B)** Tethering and fusion. **(B1)** Depicts autophagosome–lysosome fusion in the context of RAB7-associated trafficking, HOPS-mediated tethering and SNARE-dependent membrane fusion. Representative degradative SNARE machinery includes STX17, SNAP47 and VAMP7 or VAMP8 (Wang et al., 2016; Jian et al., 2024; van der Beek et al., 2024). **(B2)** Depicts fusion of a secretory carrier with the plasma membrane and soluble cargo release; SEC22B, STX3/STX4 and SNAP23/SNAP29 have been implicated in the IL-1β secretory pathway (Kimura et al., 2017). SNARE complexes are shown schematically without assigning individual strands to specific proteins or depicting exact subunit stoichiometry. Alternative assemblies have context-dependent roles and are not universally interchangeable. **(C)** Modification-state control in distinct experimental contexts. **(C1)** mTORC1 inhibition promotes GRASP55 dephosphorylation and redistribution from the Golgi to secretion-related compartments (Nüchel et al., 2021). **(C2)** Glucose deprivation reduces GRASP55 O-GlcNAcylation and supports autophagosome–lysosome fusion (Zhang et al., 2018). **(C3)** Nutrient-poor conditions favor SKP1 phosphorylation at Thr131, V-ATPase assembly and endolysosomal acidification. **(C4)** Under nutrient-rich conditions, dephosphorylated SKP1 associates with SEC22B and favors unconventional secretion from hybrid endosomal/autophagic compartments (Li et al., 2023). These GRASP55 and SKP1 modules do not form a single signaling cascade. **(D)** Context-dependent outcomes of impaired fusion. **(D1)** PTP1B-mediated STX17 dephosphorylation in an osteoblast model of prostate cancer bone metastasis reduces lysosomal fusion and increases LC3-positive EV output (Wei et al., 2024). **(D2)** *Drosophila* Syx17 deficiency produces a HOPS/Plekhm1-dependent tethering lock that impairs fusion and autophagosome secretion (Hargitai et al., 2025). The vesicle cartoons illustrate output phenotypes rather than a universally established membrane origin, LC3 topology or cargo topology. Black arrows indicate the sequence or direction of the labeled processes. Green upward and red downward arrows indicate increased and decreased activity or output, respectively, in the indicated experimental context. Dashed lines separate subpanels and do not denote regulatory interactions. P denotes phosphorylation. Proteins, complexes and membranes are schematic and not to scale. Panels show representative modules rather than a universal linear pathway. Other abbreviations are defined in the main text and abbreviation list.

**TABLE 1:** Route definitions and discriminating evidence.

Route	Definition	Diagnostic evidence	Key machinery/readouts	Key caveat	References
Degradative autophagy	Autophagosomes fuse with lysosomes; cargo is degraded	Fusion, acidification and cargo degradation; assess extracellular cargo alongside degradative flux	STX17–SNAP29 or STX17–SNAP47 with VAMP7/VAMP8 (SNARE complexes) HOPS (tethering complex) EPG5 (RAB7 effector/tether)	Extracellular cargo after lysosome blockade does not establish basal secretion	(Wang et al., 2016; Jian et al., 2024; van der Beek et al., 2024; Shinoda et al., 2024; Zheng et al., 2024; Huang et al., 2023; Huang et al., 2021; Hargitai et al., 2025; Zhang et al., 2018; Zhang et al., 2019; Laczkó-Dobos et al., 2024; Shen et al., 2021; Jian et al., 2025)
Direct secretory autophagy	Autophagy-related carriers fuse with the plasma membrane to release soluble cargo	Free extracellular cargo, plasma-membrane fusion, ER-Golgi bypass, and selective machinery dependence	IL-1β model: TRIM16 cargo receptor SEC22B with STX3/STX4 and SNAP23/SNAP29 for secretion	ATG or LC3 dependence alone cannot distinguish this route from EV release or lysosomal exocytosis	(Kimura et al., 2017; Li et al., 2022; Dupont et al., 2011; Zhang et al., 2015; Claude-Taupin et al., 2018; Zhang et al., 2020; Kim et al., 2021; Vats and Galli, 2022; Nüchel et al., 2021; Chiritoiu et al., 2019; Bel et al., 2017)
EV-associated autophagy-linked output	Autophagy machinery supports cargo loading or secretion of membrane-enclosed EVs	Membrane enclosure, EV identity and density, protease protection, and compartment origin	LC3 conjugation, ATG12-ATG3-ALIX, CD63/ALIX/syntenin/Rab, LAMP2A	LC3-dependent endosomal loading need not pass through an autophagosome. Confirm enclosure and biogenesis before using “exosomal”	(Jeppesen et al., 2019; Ostrowski et al., 2010; Larios et al., 2020; Choezom and Gross, 2022; Matsui et al., 2023; Leidal et al., 2020; Leidal and Debnath, 2021; Gardner et al., 2023; Xu et al., 2022; Murrow et al., 2015; Ferreira et al., 2022; Colombo et al., 2013; Solvik et al., 2022; Mao et al., 2025; Leidal and Debnath, 2020; Liu et al., 2023; Verweij et al., 2022; Sahu et al., 2011; Mejlvang et al., 2018; (Li et al., 2023)
Lysosomal stress-associated rerouting	Lysosomal impairment redirects degradation-bound cargo outward	Defined lysosomal defect, selective export, lysis controls, and rescue of degradative routing	Lysosomal inhibitors or fusion defects; compare increased export with HOPS-dependent trapping and reduced secretion	Stress-defined. SALI cargo may associate with EV surfaces or nanoparticles; EVP detection does not establish lumenal packaging	(Xu et al., 2022; Solvik et al., 2022; Shelke et al., 2023; Wei et al., 2024; van der Beek et al., 2024; Debnath and Leidal, 2022; Sagini et al., 2021; Hargitai et al., 2025; Guo et al., 2017; Cas et al., 2019; Hessvik et al., 2016)
Lysosomal exocytosis comparator	Lysosomes fuse with the plasma membrane and release their contents	Ca2+-regulated fusion, surface LAMP1, hydrolase release, and lysosomal origin	Surface LAMP1 and lysosomal hydrolase release	Keep distinct from secretory autophagy and EV output unless the terminal compartment is shown	(Buratta et al., 2020; Medina et al., 2011; Spampanato et al., 2013)
Unresolved autophagy-linked output	Autophagy-linked extracellular cargo without sufficient evidence to assign a route	Incomplete evidence for enclosure, compartment origin, terminal fusion, or cell integrity	LC3/ATG, CD63/LAMP1, inhibitor response, or cargo enrichment alone	Use “autophagy-linked extracellular output” until orthogonal evidence resolves the route	(Buratta et al., 2020; Leidal and Debnath, 2021; Xu et al., 2022; Mao et al., 2025; van der Beek et al., 2024)

Abbreviations: ALIX, apoptosis-linked gene 2-interacting protein X; ATG, autophagy-related; CD63, cluster of differentiation 63; EPG5, ectopic P-granules 5 autophagy tethering factor; ER, endoplasmic reticulum; EV, extracellular vesicle; EVP, extracellular vesicle and particle; HOPS, homotypic fusion and vacuole protein sorting complex; IL-1β, interleukin-1β; LAMP1/LAMP2A, lysosome-associated membrane protein 1/2A; LC3, microtubule-associated protein 1 light chain 3; RAB/Rab, Rab-family small GTPase (including RAB7); SALI, secretory autophagy during lysosome inhibition; SEC22B, SEC22 homolog B, vesicle trafficking protein; SNAP23/29/47, synaptosome-associated proteins 23/29/47; SNARE, soluble N-ethylmaleimide-sensitive factor attachment protein receptor; STX3/4/17, syntaxins 3/4/17; TRIM16, tripartite motif-containing protein 16; VAMP7/8, vesicle-associated membrane proteins 7/8.

**TABLE 2:** Cargo classes and route evidence.

Cargo class	Examples	Likely route(s)	Evidence and boundary	Key observations	References
Leaderless cytokines and danger signals	IL-1β; HMGB1	Direct secretion; other outputs depend on context	Cargo-specific machinery is resolved for IL-1β; HMGB1 uses additional trafficking steps	TRIM16/SEC22B and SNARE dependence; ER–Golgi bypass; cargo release and lysis controls	(Kimura et al., 2017; Li et al., 2022; Dupont et al., 2011; Zhang et al., 2015; Claude-Taupin et al., 2018; Zhang et al., 2020; Kim et al., 2021; Vats and Galli, 2022)
RNA-binding and transmembrane cargo	RNA-binding proteins; TFRC	EV-associated output, especially LDELS	LC3 conjugation supports loading in defined models; verify EV identity	LC3 dependence; cargo enrichment and enclosure; endosomal/EV markers	(Leidal et al., 2020; Leidal and Debnath, 2021; Gardner et al., 2023; Leidal and Debnath, 2020)
KFERQ-like EV cargo	HIF1A; KFERQ-tagged proteins	LAMP2A-dependent endosomal loading into exosomes	KFERQ-dependent loading is supported; shared chaperones do not establish lysosomal CMA	LAMP2A/HSC70 dependence; endosomal uptake; membrane-enclosed EV cargo	(Ferreira et al., 2022)
CMA-linked secretory cargo	PARK7/DJ-1	Lysosome-related unconventional secretion	Functional lysosomes support stress-induced release; EV enclosure is not established by these observations	CMA-dependent lysosomal delivery STX17 and terminal SNARE dependence	(Dash et al., 2025)
Aggregation-prone and proteostatic cargo	α-Synuclein; tau; mutant huntingtin; SOD1; UBB+1	Direct, EV-associated or stress-rerouted release	Routes and donor/recipient effects differ across cargoes and models	Autophagy/lysosome perturbation; compartment and cargo-topology evidence; functional readouts	(Nakamura et al., 2024; Minakaki et al., 2018; Ejlerskov et al., 2013; Kang et al., 2019; Ahat et al., 2022; Abe et al., 2024; Wagh and Glickman, 2025; Hutchings et al., 2024; Sawai et al., 2025; Palumbos et al., 2025)
Organelles and large cellular material	Mitochondria or their components	Stress-associated disposal, sometimes EV- or autophagy-linked	Autophagy requirements vary; avoid a shared-route inference	Organelle identity; lysis exclusion; enclosure; lysosomal status and recipient effects	(Tan et al., 2022; Liang et al., 2023)
Viral material	Poliovirus; EV71; encephalomyocarditis virus; HBV Coxsackievirus A10	EV/amphisome-associated or other non-lytic release	Carrier formation, RNA loading and infectivity are distinct endpoints	Genetic perturbation; cargo loading/enclosure; replication, lysis and recipient infectivity	(Aponte-Diaz et al., 2025; Zhang et al., 2022; Li et al., 2025; van der Grein et al., 2022; Wang R. et al., 2025)
Barrier and antimicrobial cargo	Paneth-cell lysozyme	Direct secretory-autophagy-like release	Protection is shown during infection-induced stress	Cell identity; bacterial challenge; lysozyme release and ATG dependence	(Bel et al., 2017; Cray et al., 2021)

Abbreviations: ATG, autophagy-related; CMA, chaperone-mediated autophagy; ER, endoplasmic reticulum; EV, extracellular vesicle; EV71, enterovirus 71; HBV, hepatitis B virus; HIF1A, hypoxia-inducible factor 1α; HMGB1, high mobility group box 1; HSC70, heat shock 70-kDa protein 8; IL-1β, interleukin-1β; KFERQ, lysine–phenylalanine–glutamate–arginine–glutamine motif; LAMP2A, lysosome-associated membrane protein 2A; LC3, microtubule-associated protein 1 light chain 3; LDELS, LC3-dependent EV, loading and secretion; PARK7/DJ-1, Parkinsonism-associated deglycase, also known as DJ-1; SEC22B, SEC22 homolog B, vesicle trafficking protein; SNARE, soluble N-ethylmaleimide-sensitive factor attachment protein receptor; SOD1, superoxide dismutase 1; STX17, syntaxin 17; TFRC, transferrin receptor; TRIM16, tripartite motif-containing protein 16; UBB+1, frameshift-derived ubiquitin B variant.

Entries describe model-specific observations and boundaries rather than a formal evidence-grading scale.

## 3 Molecular regulation of degradative and secretory fates

Multiple routes do not imply random selection. Cellular state, fusion competence, tethering, and stress-induced rerouting influence compartment fate. Degradative fusion is the reference state, but its disruption has different consequences across experimental systems. Cargo may be secreted in one setting and trapped in non-productive intermediates in another. The position and nature of the block are therefore as important as the loss of degradation itself.

### 3.1 The degradative baseline

Under homeostatic conditions, the autophagosome is generally biased toward degradative fusion. STX17-centered fusion machinery, HOPS recruitment, and lysosomal delivery establish a baseline state in which cargo is consumed rather than externalized (Jian et al., 2024; Huang et al., 2021). This distinction matters because secretory phenotypes are best read against an actively maintained degradative baseline rather than as constitutive parallel outputs of equal weight.

### 3.2 Lysosomal dysfunction as a fate redirector

The strongest evidence for stress-induced fate redirection comes from defined lysosomal perturbations. SALI denotes selective export of autophagy-linked cargo during lysosomal inhibition, rather than physiological secretion in general (Solvik et al., 2022; Debnath and Leidal, 2022). In serum-starved HEK293T cells, Solvik et al. tested 20 nM bafilomycin A1 or 25 μM chloroquine for 16 h and detected increased extracellular LC3-II and cargo receptors in extracellular vesicle and particle (EVP) fractions (Solvik et al., 2022). Supplementary experiments showed related cargo-receptor release in mouse R221A and human MDA-MB-231 breast-cancer cells and mouse Neuro2a and human SH-SY5Y neuronal cell lines treated with 20 nM bafilomycin A1 for 18 h in serum-free medium (Solvik et al., 2022). These concentrations and durations define the reported experiments; they do not constitute a common stress threshold across cell types.

The extracellular form of this material is an important boundary. In the SALI study, the cargo receptors p62, NBR1 and optineurin (OPTN) in EVP fractions were largely susceptible to trypsin without detergent, and immunogold microscopy localized p62 to EV surfaces or extracellular nanoparticles rather than the vesicle lumen (Solvik et al., 2022). Thus, co-isolation with EVs should not be rewritten as intravesicular packaging. The same study used genetic disruption of autophagosome formation or release to establish pathway dependence; this evidence distinguishes the response from passive leakage, although cell-integrity controls remain necessary under each new exposure condition (Solvik et al., 2022). Chloroquine- and other lysosome-impairment studies also reported autophagy-associated extracellular material, but their marker profiles do not establish one common cargo topology or terminal route (Xu et al., 2022; Sagini et al., 2021).

The available evidence therefore supports a conditional response to impaired lysosomal processing, not a universal conversion of degradation into secretion. Cholesterol-deficient cellular models provide another defined setting in which autophagosome-dependent small-EV release increased, including models of cholesterol-biosynthesis disorders and head and neck cancer (Yaeger et al., 2026). This lipid-dependent response should retain its own experimental context rather than being equated with pharmacological SALI. Fusion defects can also suppress secretion, as the tethering-lock example in Section 3.3 illustrates (Hargitai et al., 2025). Interpretation therefore requires the defect, cargo, remaining trafficking capacity and exposure conditions to be specified.

### 3.3 Active molecular switches and upstream biasing signals

A more direct form of fate control appears in switch-like mechanisms that act on fusion specificity itself. A well-resolved route-biasing example is the protein tyrosine phosphatase 1B (PTP1B)–STX17 axis. In a prostate cancer bone metastasis model, PTP1B-mediated dephosphorylation of STX17 reduced autophagosome-lysosome fusion and redirected cells toward secretory autophagy, producing LC3-positive extracellular vesicles that contributed to osteogenic remodeling (Wei et al., 2024). The study linked a defined post-translational event to a measurable shift in route usage and supports the idea that autophagosomal fate can be rewired through a molecular switch in a disease context (Wei et al., 2024). Impaired STX17 function does not, however, uniformly increase secretion. In *Drosophila*, Syntaxin17 loss trapped autophagosomes and lysosomes in stable clusters, blocking endolysosomal progression and autophagosome secretion (Hargitai et al., 2025). This tethering lock depended on HOPS and its adaptor, pleckstrin homology domain-containing family M member 1 (PLEKHM1). Considered together, these studies indicate that fusion failure can either bias secretion or produce a non-productive tethered intermediate, depending on the state of the downstream fusion machinery.

Nutrient and stress signaling regulate GRASP55 through distinct modifications and binding partners. In unstressed cells, mechanistic target of rapamycin complex 1 (mTORC1) phosphorylates GRASP55 and retains it at the Golgi. Stress-induced mTORC1 inactivation promotes GRASP55 dephosphorylation and redistribution to compartments supporting unconventional secretion (Nüchel et al., 2021). Glucose deprivation engages a different mechanism. Reduced O-GlcNAc modification of GRASP55 permits its association with LC3-II and LAMP2, the lysosome-associated membrane protein 2, to facilitate autophagosome–lysosome fusion (Zhang et al., 2018). During amino acid starvation, GRASP55 supports degradative fusion through beclin 1 (BECN1) and the UV radiation resistance-associated protein (UVRAG)-containing phosphatidylinositol 3-kinase complex (Zhang et al., 2019). These studies distinguish regulation of secretory output from support of degradative fusion; GRASP55 redistribution alone does not identify the terminal route.

GRASP-family proteins also participate in distinct unconventional export models, including developmental AcbA secretion in *Dictyostelium* (Kinseth et al., 2007) and ΔF508-CFTR trafficking (Gee et al., 2011). In stimulated macrophages, GRASP55 and inositol-requiring enzyme 1α (IRE1α), an endoplasmic-reticulum stress sensor, help maintain mature IL-1β in a secretion-competent state (Chiritoiu et al., 2019). These cargo-specific observations should be distinguished from the fusion-regulatory roles described above.

S-phase kinase-associated protein 1 (SKP1) is an assembly factor in ubiquitin-ligase complexes, with a separate role in endolysosomal trafficking (Zubkova et al., 2024; Li et al., 2023). Under nutrient-poor conditions, phosphorylation of SKP1 at Thr131 promotes assembly of the vacuolar ATPase proton pump and endolysosomal acidification. Under nutrient-rich conditions, dephosphorylated SKP1 interacts with SEC22B and favors secretion from less acidified hybrid endosomal/autophagic compartments (Li et al., 2023). This nutrient-dependent mechanism links a defined modification state to compartment acidification and secretory readiness. Together with the ATG5-dependent branching described earlier, it illustrates how fate regulation can act upstream of terminal membrane fusion (Wang et al., 2023; Guo et al., 2017).

### 3.4 A decision matrix rather than a single pathway

Current evidence is better organized as a decision matrix than as a single linear switch. Lysosomal competence determines whether degradative processing remains available (Solvik et al., 2022; Shelke et al., 2023). Fusion machinery and its modification state can directly bias terminal destination, as illustrated by STX17-centered control (Wei et al., 2024; Jian et al., 2024; Shinoda et al., 2024; Laczkó-Dobos et al., 2024). Tethering state matters as well, because stable HOPS-dependent intermediates can trap cargo and block both degradation and secretion (van der Beek et al., 2024; Hargitai et al., 2025). Nutrient and stress signaling add a further layer of bias through modules such as GRASP55 and SKP1 (Nüchel et al., 2021; Zhang et al., 2018; Zhang et al., 2019; Li et al., 2023). Extracellular output from autophagy-related membranes is therefore best treated as the product of layered regulatory inputs rather than as a single secretory autophagy pathway. Figure 2 illustrates representative molecular modules underlying these decisions and their context-dependent outcomes; Table 1 summarizes the route definitions and caveats.

## 4 Physiological deployment of autophagy-linked extracellular output

Autophagy-linked extracellular output can support regulated signaling and host defense, although a protective function does not imply an absence of cellular stress. Immune stimulation, bacterial challenge and neuronal activity recruit different secretory programs. Their physiological contribution and the resolution of their terminal route should therefore be evaluated separately. Table 3 distinguishes these contexts from the disease-associated effects summarized in Table 4.

**TABLE 3:** Physiological deployment and protective responses.

Context and cells	Cargo and route evidence	Function and boundary	References
Regulated immune signaling	IL-1β: TRIM16/SEC22B-dependent secretion HMGB1: autophagy-linked and endolysosomal trafficking	Controlled inflammatory communication; exclude cell-death-associated release The two cargoes need not share one terminal route	(Kimura et al., 2017; Li et al., 2022; Dupont et al., 2011; Zhang et al., 2015; Claude-Taupin et al., 2018; Zhang et al., 2020; Kim et al., 2021; Vats and Galli, 2022)
Infected intestinal Paneth cells	Lysozyme rerouting through secretory autophagy during bacterial challenge	Preserves antimicrobial defense when conventional secretion is impaired; protective, but induced by infection and ER stress	(Bel et al., 2017)
Activity-stimulated neurons	α-Synuclein release involving ATG5, BECN1 and RAB8A in neuronal models	Regulated extracellular release; activity dependence does not establish beneficial recipient signaling or pathological spread	(Nakamura et al., 2024)
Glucose-stimulated β cells	Insulin secretion involving autophagy machinery and active RAB37	Regulated insulin output in cell and mouse models; not evidence for a general EV route or a SALI threshold	(Wu et al., 2023)

Abbreviations: ATG5, autophagy-related protein 5; BECN1, beclin 1; ER, endoplasmic reticulum; EV, extracellular vesicle; HMGB1, high mobility group box 1; IL-1β, interleukin-1β; RAB8A/RAB37, Ras-related proteins Rab-8A/Rab-37; SALI, secretory autophagy during lysosome inhibition; SEC22B, SEC22 homolog B, vesicle trafficking protein; TRIM16, tripartite motif-containing protein 16.

**TABLE 4:** Pathological dysregulation and therapeutic implications.

Disease and model	Cargo/route and consequence	Therapeutic implication and boundary	References
Neurodegenerative/proteostatic models	α-Synuclein, tau, UBB+1 and SOD1 use heterogeneous direct, lysosome-related or EV-associated outputs; disposal and extracellular injury can coexist	Test restoration of cargo clearance alongside release inhibition. Measure intracellular burden and recipient injury: reduced SOD1 secretion produced divergent onset and survival effects	(Minakaki et al., 2018; Ejlerskov et al., 2013; Kang et al., 2019; Ahat et al., 2022; Abe et al., 2024; Wagh and Glickman, 2025; Hutchings et al., 2024)
Neuroinflammation; mouse and *postmortem* human brain	SKA2 restrains FKBP51-associated IL-1β secretion. Ska2 loss promotes an inflammatory cycle and degeneration in mice	Candidate selective control of inflammatory cargo release; human associations do not establish treatment efficacy	(Hartmann et al., 2024)
Prostate cancer bone-metastasis models	Tumor EVs activate osteoblastic PTP1B–STX17 fate redirection and LC3-positive EV output, supporting abnormal bone remodeling	Interrupt cross-cellular reprogramming or restore degradative fusion; genetic evidence is preclinical and cell-specific	(Wei et al., 2024)
Glioblastoma during temozolomide treatment	Autophagy-linked HMGB1 release promotes macrophage M1-like polarization and chemosensitivity	Preserve beneficial treatment-induced communication where supported; blanket secretion inhibition could oppose the observed response	(Li et al., 2022)
Hypoxic oral-cancer stromal models	Fibroblast serglycin secretion promotes matrix remodeling and invasion through a route shift from conventional secretion	Target pathological stromal output; distinguish cargo-specific effects from broad autophagy inhibition	(Zhang et al., 2025; New et al., 2017)
Viral infection models	Poliovirus 3CD affects carrier formation/loading; EV71 RAB11-dependent trafficking supports RNA loading. Other viruses use distinct EV- or autophagy-associated outputs	Separate genome replication, particle loading, non-lytic release and infectivity when evaluating candidate antiviral effects. No class-wide clinical validation	(Aponte-Diaz et al., 2025; Zhang et al., 2022; Li et al., 2025; van der Grein et al., 2022; Wang R. et al., 2025)
Vascular inflammation; endothelial-cell and mouse experiments	Autophagy-related KARS1 release with externally exposed EV-associated cargo; proposed amphisome involvement remains incompletely resolved	Explore cargo-specific extracellular signaling. *In vitro* effects were not reproduced *in vivo*; no validated vascular therapy follows	(Yun et al., 2025)

Abbreviations: 3CD, poliovirus nonstructural 3C–3D precursor protein; EV, extracellular vesicle; EV71, enterovirus 71; FKBP51, FK506-binding protein 51; HMGB1, high mobility group box 1; IL-1β, interleukin-1β; KARS1, lysyl-tRNA, synthetase 1; LC3, microtubule-associated protein 1 light chain 3; PTP1B, protein tyrosine phosphatase 1B; RAB11, Rab11-family small GTPase; SKA2, spindle and kinetochore-associated complex subunit 2; SOD1, superoxide dismutase 1; STX17, syntaxin 17; UBB+1, frameshift-derived ubiquitin B variant.

### 4.1 Immune signaling

Immune signaling is one of the best-characterized physiological contexts for secretory autophagy. Interleukin-1β remains a well-established example of autophagy-linked unconventional secretion. Early work showed that IL-1β can access a vesicular intermediate linked to autophagy and be exported independently of classical ER-Golgi trafficking (Dupont et al., 2011; Zhang et al., 2015; Claude-Taupin et al., 2018). Subsequent mechanistic studies identified specialized secretory machinery, including TRIM16, SEC22B, and plasma membrane SNARE components, supporting the view that IL-1β export reflects an organized route rather than incidental leakage (Kimura et al., 2017; Noh et al., 2022).

HMGB1 broadens this logic. Although it differs from IL-1β in mechanistic detail, HMGB1 secretion also draws on secretory autophagy machinery and endolysosomal trafficking, with dependence on the chaperone heat shock protein 90 alpha family class A member 1 (HSP90AA1), Golgi reassembly stacking protein 2 (GRASP55/GORASP2), ATG factors, MVB-linked processes, and Rab proteins (Li et al., 2022; Kim et al., 2021). These two cargoes together show that autophagy-related secretion can participate in controlled inflammatory communication (Cavalli and Cenci, 2020).

### 4.2 Barrier and antimicrobial defense

Barrier defense illustrates protective secretion during stress. In infected intestinal Paneth cells, bacteria-induced endoplasmic reticulum stress and disrupted conventional secretion rerouted lysozyme through secretory autophagy, limiting bacterial dissemination (Bel et al., 2017). This was an infection-induced defense response, rather than constitutive secretion in unstressed tissue. It provides *in vivo* evidence for beneficial extracellular output but does not establish that Paneth cells use the same lysosomal-inhibition mechanism as SALI (Solvik et al., 2022; Bel et al., 2017).

### 4.3 Activity-dependent neuronal communication

Regulated release also occurs in activity-dependent neuronal communication. In mammalian neurons, neuronal activity and cytosolic Ca2+ elevation promote ATG5-, BECN1-, and RAB8A-dependent secretion of α-synuclein through an autophagosomal route in which α-synuclein associates with LC3-positive structures rather than LAMP1-positive lysosomes (Nakamura et al., 2024). A published account of *Drosophila* work also links secretory autophagy to activity-induced synaptic remodeling (Chang and Chang, 2024). These studies are consistent with the principle that activity-dependent extracellular output can be an organized physiological use of autophagy-related membranes rather than a byproduct of failed degradation (Veh and Lüningschrör, 2026).

These cases show regulated autophagy-linked release during immune activation, barrier defense and neuronal activity, with different levels of evidence for its downstream function. Activity-dependent release alone does not establish beneficial recipient signaling. The functional consequence must therefore be tested alongside the route, as summarized in Table 3.

Regulated secretion also provides a counterpoint to the lysosomal-inhibition experiments. Glucose-stimulated insulin secretion involved active RAB37 and autophagy machinery in β-cell and mouse models (Wu et al., 2023), whereas neuronal activity stimulated autophagy-linked α-synuclein release in primary cortical neurons and SH-SY5Y cells (Nakamura et al., 2024). These studies support stimulus- and cell-specific deployment of secretory machinery; they do not establish that physiological secretion requires the lysosomal lesion or the exposure conditions used to define SALI.

## 5 Pathological dysregulation of autophagosomal fate

Disease examples are more informative when grouped by mechanism rather than diagnosis. We distinguish hyperactivation of secretory output, proteostatic rerouting after degradative stress or cargo overload, and microenvironmental reprogramming of fate decisions in neighboring cells. This scheme is intended for analysis, not as an exhaustive disease classification, and each assignment depends on the available route evidence.

### 5.1 Hyperactivation of secretory output

One well-characterized example of hyperactivation comes from neuroinflammation. Spindle and kinetochore-associated complex subunit 2 (SKA2) was identified as a brake on secretory autophagy-dependent IL-1β release, acting by antagonizing the co-chaperone FK506-binding protein 51 (FKBP51, encoded by FKBP5) and limiting assembly of a secretory SNARE complex involving SEC22B, SNAP29, and syntaxin-related components (Hartmann et al., 2024). In this setting, secretory autophagy does not simply accompany inflammation but amplifies it. Loss of SKA2 enhanced IL-1β release, promoted inflammasome-linked neurotoxicity, and generated a feedforward cycle of inflammatory damage (Hartmann et al., 2024).

Hippocampal Ska2 knockdown in mice caused marked neuroinflammatory and neurodegenerative phenotypes. Analyses of human *postmortem* brain tissue also found increased secretory-autophagy markers in Alzheimer’s disease (Hartmann et al., 2024). These data support a link between hyperactivated secretory autophagy and neuroinflammation-associated degeneration, but they do not establish a universal mechanism across neurodegenerative diseases. This study remains a strong example of excessive secretory output caused by loss of a defined molecular brake.

Vascular inflammation offers a distinct, less route-resolved example. In endothelial-cell experiments, calcium and starvation stimulated autophagy-related secretion of lysyl-tRNA synthetase 1 (KARS1), an aminoacyl-tRNA synthetase with extracellular inflammatory activity (Yun et al., 2025). Secreted KARS1 was externally exposed in exosome-associated fractions, and the proposed amphisome route remains partly inferential. Moreover, the authors reported that the *in vitro* findings were not reproduced in their mouse experiments (Yun et al., 2025). This evidence supports further study of cargo-specific vascular signaling, but not a validated anti-atherosclerotic intervention.

### 5.2 Proteostatic rerouting after degradative stress

Another form of dysregulation occurs when degradative competence fails or proteotoxic cargo is rerouted outward under stress. In neurons, autophagy inhibition and lysosomal dysfunction can promote extracellular release of pathogenic proteins in vesicles with hybrid autophagosome-exosome-like properties (Minakaki et al., 2018). Impaired autophagosome-lysosome fusion likewise promotes unconventional α-synuclein secretion, suggesting that failure of the degradative gate can externalize material that would otherwise remain intracellular (Ejlerskov et al., 2013). Autophagy-dependent secretion has also been reported for both normal and pathological tau in neuronal models (Kang et al., 2019). GRASP55-dependent unconventional secretion has been implicated in mutant huntingtin export (Ahat et al., 2022). In macrophage-lineage cells, lysosomal overload drives exosomal release of insoluble, seed-competent α-synuclein through the leucine-rich repeat kinase 2 (LRRK2)–RAB10 pathway, indicating that extracellular propagation depends on both cell type and cargo state (Abe et al., 2024).

Not all disease-linked secretion in this group is passive spillover from lysosomal failure. The Alzheimer-associated ubiquitin variant UBB+1 is recognized by SQSTM1 or p62, loaded into autophagosomes, and secreted through SEC22B-STX4-SNAP23-mediated direct secretory autophagy (Wagh and Glickman, 2025). Lysosomal inhibition only partially stabilizes the intracellular cargo, which suggests that degradation and secretion can act as complementary disposal routes. In motor neurons, pleckstrin homology domain-containing family member 5 (PLEKHG5) acts as a guanine nucleotide exchange factor that activates RAB26. This pathway exports superoxide dismutase 1 (SOD1) through fusion of autophagosomal carriers with lysosome-related secretory organelles (Hutchings et al., 2024). In SOD1G93A mice, Plekhg5 deletion reduced secretion and increased presynaptic SOD1 accumulation; disease onset accelerated, whereas survival was prolonged alongside attenuated microglial activation (Hutchings et al., 2024). Thus, intracellular disposal and extracellular inflammatory effects can favor different intervention outcomes.

These studies reveal several forms of extracellular disposal under proteostatic stress, alongside the experimentally defined SALI response. Direct *in vivo* proof that one shared secretory-autophagy route drives human protein spreading remains limited (Solvik et al., 2022; Minakaki et al., 2018; Dash et al., 2025; Sawai et al., 2025). Chronic neuronal stress may instead activate multiple compensatory outputs rather than one canonical program, as suggested by the distinct secretory responses seen in stressed neurons (Palumbos et al., 2025). These models show that proteostatic stress can promote extracellular disposal. The route, subsequent spread and recipient-cell injury depend on cargo identity and cellular context (Abe et al., 2024; Wagh and Glickman, 2025; Hutchings et al., 2024).

### 5.3 Microenvironmental hijacking of fate decisions

A distinct form of dysregulation is microenvironmental hijacking, in which 1 cell population rewires the fate decisions of another. Cancer currently provides some of the most route-resolved examples. In prostate cancer bone metastasis, tumor-derived extracellular vesicles induced PTP1B-dependent dephosphorylation of STX17 in osteoblasts, reducing degradative fusion and biasing the cells toward secretory autophagy-associated LC3-positive extracellular vesicle output (Wei et al., 2024). This is a qualitatively different form of dysregulation from the intracellular failures described above: the tumor does not merely exploit its own secretion but reprograms the fate choice of a neighboring cell population (Wei et al., 2024).

Other tumor contexts involve different cargoes and secretory mechanisms. In glioblastoma, temozolomide-induced HMGB1 release through secretory autophagy promoted macrophage M1-like polarization and increased chemosensitivity (Li et al., 2022). Secretory output may therefore influence treatment response as well as tumor progression. Hypoxia also alters stromal secretion in oral squamous cell carcinoma-associated fibroblasts. Under these conditions, serglycin switches from the conventional ER-Golgi route to autophagosome-dependent, lysosome-independent secretion, promoting extracellular matrix remodeling and invasion through interactions with matrix metalloproteinases 2 and 9 (MMP2 and MMP9) (Zhang et al., 2025). The consequences of microenvironmental reprogramming therefore depend on the cargo, recipient cell, and disease context.

Other tumor studies offer less route-resolved evidence that autophagy-related proteins influence EV production or cargo loading. ATG5 can regulate exosome production independently of canonical macroautophagy in metastatic settings (Guo et al., 2017). GABA type A receptor-associated protein-like 1 (GABARAPL1), an LC3/GABARAP-family member, supports endosomal maturation, cargo sorting and pro-angiogenic EV release under hypoxia (Keulers et al., 2021). Additional tumor models implicate stress-linked amphisome or EV release (Hao et al., 2021; Li et al., 2021; Xi et al., 2021; Jiang et al., 2023). The most detailed evidence for fate control comes from PTP1B-STX17 signaling, cargo-defined secretory pathways, and hypoxia-induced stromal route switching (Li et al., 2022; Wei et al., 2024; Zhang et al., 2025). Other cited tumor studies broaden the biological context but do not resolve the route to the same extent (Zheng et al., 2026; New et al., 2017; Ly et al., 2025).

### 5.4 Pathogen exploitation

Pathogen exploitation can involve cargo selection as well as terminal release. In poliovirus-infected HeLa cells, a phenylalanine-to-tyrosine substitution in a putative LC3-interacting region (LIR) of the viral nonstructural protein 3CD impaired virion incorporation into LC3-positive carriers, trafficking to the cell surface and non-lytic spread (Aponte-Diaz et al., 2025). The mutant also disrupted normal autophagosome biogenesis. These results implicate 3CD in carrier formation and loading; they should not be reduced to proof of a single, isolated LC3-binding defect or generalized to all picornaviruses.

Enterovirus 71 (EV71) illustrates a different level of control. In infected 293T cells, RAB11 knockdown reduced viral RNA per exosome without reducing intracellular viral replication; imaging supported an autophagosome–MVB trafficking step (Zhang et al., 2022). The study distinguished viral RNA packaging from exosome biogenesis. Depletion of the RNA-associated proteins GW182 or Argonaute 2 (AGO2) reduced vesicular miR-146a and the replication capacity of transferred RNA in recipient cells, without reducing viral RNA packaging itself (Zhang et al., 2022). Thus, vesicle abundance, genome loading and infectivity are separate readouts. Neither this study nor the poliovirus work establishes a clinically effective host-directed antiviral strategy.

Other examples include encephalomyocarditis virus release, hepatitis B virus production and Coxsackievirus A10 spread after fusion blockade (Li et al., 2025; van der Grein et al., 2022; Wang R. et al., 2025). These findings should remain virus-specific: disrupting host autophagy or EV release may affect different stages of infection, so reduced extracellular virus alone cannot identify the inhibited step. Candidate interventions should distinguish effects on intracellular replication, carrier loading, cell lysis and recipient-cell infection, as the poliovirus and EV71 studies illustrate (Aponte-Diaz et al., 2025; Zhang et al., 2022).

### 5.5 Disease-specific implications for intervention

Across diseases, the shared therapeutic question is which cargo should be retained, degraded or released, and in which cell. Even within cancer, the direction of benefit can differ: the RASAL2-knockout breast-cancer model discussed in Section 6.2 warns that reducing a secretory output can restore tumor growth. Tumor-associated secretion can promote bone-niche remodeling yet also enhance temozolomide responses through HMGB1-dependent immune communication (Li et al., 2022; Wei et al., 2024). In neurodegeneration, reducing SOD1 export can worsen intracellular accumulation while changing inflammatory injury and survival (Hutchings et al., 2024). Inflammatory IL-1β release and viral carrier loading identify still different intervention points (Hartmann et al., 2024; Aponte-Diaz et al., 2025; Zhang et al., 2022). Table 4 compares these disease-specific consequences and candidate directions; Table 5 addresses the separate question of pharmacological evidence and translational maturity.

**TABLE 5:** Pharmacological interventions and candidate nodes at the secretory autophagy–EV interface.

Strategy	Intervention/node	Interface effect and directness	Model and functional result	Evidence maturity	Limitation/next step; references
Restore degradation	C1; TFEB-associated activation	TFEB nuclear localization and lysosomal restoration; direct binding not established here	Cholangiocarcinoma cells and mouse liver-metastasis models; 6-week oral gavage reduced metastatic burden	Preclinical pathway-level intervention	Connect exposure to TFEB-dependent cargo redirection. Human PTEN/CD63 associations provide context, not C1 efficacy (Jiang et al., 2023).
Restore degradation	Asteltoxin; mitochondrial ATP synthase	ATP reduction → AMPK/mTORC1 → MiT/TFE lysosomal programs; indirect EV effect	Cultured cancer cells: reduced EV release and altered MVB/lysosome balance	Preclinical pharmacological tool	Define separation from metabolic toxicity; establish *in vivo* exposure and disease benefit (Mitani et al., 2022).
Block secretion	SAR405 VPS34/class III PI3K	Defined kinase inhibitor affecting autophagic and endosomal traffic; not secretory-route selective	Head and neck cancer/CAF xenografts: local SAR405 plus cisplatin reduced tumor growth	Preclinical drug intervention	50 μL of 10 μM SAR405 intratumorally, 5 days/week; systemic exposure and stromal delivery remain to be established (New et al., 2017).
Block secretion	Zoledronic acid; FPPS/prenylation pathway	RAB7 prenylation and autolysosomal processing affected; no direct RAB7 targeting claim	Osteoclast models: secretory protein release and bone resorption reduced	Clinical medicine; proposed secretory mechanism remains preclinical	Establish contribution of this route to benefit; assess normal bone remodeling. EV route not shown (Kim et al., 2026).
Block secretion	TRIM16 CD147–TRIM56–GCN2	Cargo receptor and regulatory signaling nodes in distinct models; not a common complex or equivalent inhibition targets	Head and neck CAF and NSCLC models: genetic perturbations link secretion to tumor phenotypes	Preclinical genetic evidence	Translate genetic dependencies into selective pharmacology; verify the direction of each perturbation (Ly et al., 2025; Yang et al., 2026)
Secretion-blockade risk	RASAL2 loss with additional RAB27A knockdown	RAB27A knockdown reduced the autophagy-associated exosome output increased by RASAL2 loss	RASAL2-knockout MDA-MB-231 cells and xenografts: additional RAB27A knockdown reversed tumor suppression	Preclinical genetic counterexample	Supports a risk of blocking secretion in this knockout background, not general benefit from RAB27A inhibition (Wang X. et al., 2025).
Block secretion	SEC22B-dependent MCT4 trafficking	Transporter localization and lactate export; not EV encapsulation of lactate	TNBC cells and mouse metastasis models: pathway linked to lactate export and progression	Preclinical candidate trafficking node	Separate transporter activity from trafficking; establish selective pharmacology (Wu et al., 2026)
Exploit coupling/restore degradation	Temsirolimus; mTOR	Autophagy-dependent MVB degradation reduces small-EV-associated PD-L1	Tumor models: improved T-cell response and anti-PD-L1 activity	Clinical medicine; EV mechanism preclinical	Separate EV contribution from systemic mTOR effects; validate cargo biomarker (Park et al., 2022)
Exploit coupling	Icaritin; aldolase B (ALDOB)/syntaxin 16 (STX16) pathway	ALDOB and STX16 expression reduced; EV transfer of autophagosomal material. No direct STX16 binding claim	HCC cell/macrophage and mouse models: recipient STAT3 degradation and tumor suppression	Preclinical evidence for this intercellular mechanism	Resolve cargo packaging and recipient specificity; assess other immune cells and toxicity (Zheng et al., 2026)

Abbreviations: AMPK, AMP-activated protein kinase; C1, curcumin analog C1; CAF, cancer-associated fibroblast; CD147, cluster of differentiation 147; CD63, cluster of differentiation 63; EV, extracellular vesicle; FPPS, farnesyl pyrophosphate synthase; GCN2, general control nonderepressible kinase 2; HCC, hepatocellular carcinoma; MCT4, monocarboxylate transporter 4; MiT/TFE, microphthalmia family of basic helix-loop-helix-leucine-zipper transcription factors; mTOR, mechanistic target of rapamycin; mTORC1, mechanistic target of rapamycin complex 1; MVB, multivesicular body; NSCLC, non-small-cell lung cancer; PD-L1, programmed death ligand 1; PI3K, phosphatidylinositol 3-kinase; PTEN, phosphatase and tensin homolog; RAB7/RAB27A, Ras-related proteins Rab-7/Rab-27A; RASAL2, Ras protein activator like 2; SEC22B, SEC22 homolog B, vesicle trafficking protein; STAT3, signal transducer and activator of transcription 3; TFEB, transcription factor EB; TNBC, triple-negative breast cancer; TRIM16, tripartite motif-containing protein 16; TRIM56, tripartite motif-containing protein 56; VPS34, vacuolar protein sorting 34.

Categories distinguish pharmacological interventions, pathway-level effects and genetic candidates; they are not a validated grading scale. Clinical medicine status does not validate the proposed interface mechanism. Strategy overlap is intentional; the RASAL2 row is a counterexample to secretion blockade, not a fourth treatment strategy. References correspond to the manuscript bibliography.

## 6 Pharmacological and translational opportunities at the secretory autophagy–EV interface

The pharmacological objective is to change the extracellular exposure of a disease-relevant cargo while preserving intracellular clearance and beneficial communication. This requires distinguishing restoration of lysosomal competence, inhibition of pathological secretion, and selective exploitation of autophagy–EV coupling. The studies discussed below support these strategies at different levels: a drug can affect a defined molecular target, alter a broader trafficking pathway, or reveal a phenotype that is also supported by genetic perturbation. None of these observations alone establishes selective pharmacological control of the secretory autophagy–EV interface in patients. Table 5 compares representative interventions and candidate nodes using these distinctions; the categories are descriptive rather than a validated evidence-grading system.

### 6.1 Restoring lysosomal competence and redirecting compartment fate

Restoring degradation is attractive when pathological extracellular output follows a demonstrable lysosomal defect. In cholangiocarcinoma models deficient in phosphatase and tensin homolog (PTEN), a lipid and protein phosphatase, the curcumin analog C1 promoted transcription factor EB (TFEB) nuclear localization, restored lysosomal function, reduced exosome secretion, and restrained metastatic phenotypes (Jiang et al., 2023). This supports a TFEB-associated route-redirection strategy, without establishing that every affected vesicle originates from a secretory autophagosome. Asteltoxin provides a different pharmacological entry point: inhibition of mitochondrial ATP synthase lowers cellular ATP and engages AMP-activated protein kinase (AMPK)–mTORC1 signaling and lysosomal transcriptional programs of the microphthalmia family of basic helix-loop-helix-leucine-zipper transcription factors (MiT/TFE), with prominent nuclear redistribution of TFE3 in the tested cells and reduced EV output (Mitani et al., 2022). Its proximal action is metabolic, so it should not be described as a selective inhibitor of EV release or secretory autophagy.

The potential advantage is simultaneous improvement of intracellular clearance and reduction of harmful extracellular cargo. However, greater lysosomal capacity need not produce less secretion in every context: TFEB can also stimulate lysosomal exocytosis (Medina et al., 2011). Likewise, evidence that TFEB activation improves clearance in storage-disease models supports degradative capacity, but does not independently demonstrate suppression of a disease-causing EV route (Spampanato et al., 2013; Palmieri et al., 2017). Translation therefore requires paired measurements of degradative flux and extracellular cargo, with lysosomal exocytosis assessed separately. The C1 study included oral gavage for 6 weeks in a mouse liver-metastasis model and human PTEN/CD63 associations (Jiang et al., 2023). These provide *in vivo* intervention and human disease-context evidence, respectively; the patient associations do not demonstrate a response to C1. Development should connect effective exposure to TFEB-dependent cargo redirection; for asteltoxin, the separation between EV modulation and energy-related toxicity is a central development question. These are proposed validation requirements, not evidence of an established therapeutic window.

### 6.2 Blocking pathological secretory output

Inhibiting a defined secretory dependency may be preferable when extracellular cargo actively sustains disease. In head and neck cancer models, autophagy inhibition in cancer-associated fibroblasts reduced tumor-promoting secretion, and the VPS34 inhibitor SAR405 enhanced cisplatin efficacy in xenografts (New et al., 2017). VPS34 is the class III phosphatidylinositol 3-kinase involved in autophagic and endosomal membrane traffic; its pharmacological tractability does not confer selectivity for the secretory branch. SAR405 was administered by intratumoral injection of 50 μL at 10 μM, 5 days per week, with cisplatin at 3 mg/kg intraperitoneally once weekly (New et al., 2017). This local regimen supports a combination-testing rationale, while leaving systemic exposure, delivery to stromal cells and route-specific target engagement to be established.

Trafficking regulators offer more context-specific mechanistic candidates. TRIM16-dependent stromal secretion has genetic support, but a selective drug corresponding to this cargo-receptor function has not been established in the cited study (Ly et al., 2025). In non-small-cell lung cancer, CD147, a type I transmembrane glycoprotein, suppressed expression of the E3 ubiquitin ligase TRIM56, a tripartite-motif-family protein. This reduced degradation of general control nonderepressible kinase 2 (GCN2) and supported the autophagy-linked exosome output examined in that model (Yang et al., 2026). CD147, TRIM56 and GCN2 therefore denote a regulatory relationship among distinct proteins, not a single protein complex or three equivalent inhibition targets.

Zoledronic acid illustrates why the maturity of a medicine and the maturity of an interface mechanism must be reported separately. A study in osteoclast models found that zoledronic acid suppressed secretory autophagy alongside bone-resorptive activity (Kim et al., 2026). Its established pharmacology involves farnesyl pyrophosphate synthase inhibition and impaired small-GTPase prenylation; the study linked altered RAB7 prenylation to defective autolysosomal processing. Thus, it is not a direct RAB7 inhibitor. Its clinical anti-resorptive use does not show that selective blockade of secretory autophagy accounts for patient benefit, and these findings do not establish an EV-mediated mechanism (Kim et al., 2026).

Cargo trafficking can also connect this interface to metabolite transport. In triple-negative breast cancer models, epidermal growth factor promoted SEC22B-dependent trafficking of the monocarboxylate transporter MCT4, encoded by SLC16A3, and lactate export associated with metastatic progression (Wu et al., 2026). This identifies a transport-related intervention hypothesis; it does not imply that lactate is encapsulated within EVs or provide evidence about drug metabolism. A useful next test would separate MCT4 transport activity from its secretory trafficking and determine which is required for the recipient-cell phenotype.

The advantage of secretion blockade is reduced exposure of neighboring cells to a harmful cargo, but its liability is loss of protective disposal or beneficial secretion. This concern is supported by protective neuronal cargo handling and Paneth-cell antimicrobial secretion (Bel et al., 2017; Hutchings et al., 2024). In addition, autophagy inhibition can increase extracellular output. In a breast-cancer model, autophagy blockade increased small-EV release of human epidermal growth factor receptor 2 (HER2), an ErbB-family receptor tyrosine kinase. Tumorigenesis decreased in this setting (Hao et al., 2021). A change in total EV number therefore cannot substitute for a cargo-specific functional endpoint. Candidate inhibitors should be tested with genetic rescue, viability controls, intracellular cargo measurements, and recipient-cell assays before their effects are attributed to selective secretion blockade.

The RASAL2 model provides a tumor-specific warning against assuming that reduced secretion is beneficial. RASAL2 (Ras protein activator like 2) belongs to the RAS GTPase-activating protein family; RAB27A is a distinct small GTPase involved in vesicle release. In RASAL2-knockout MDA-MB-231 cells, autophagy-associated exosome secretion increased while proliferation, migration and xenograft growth decreased. Additional RAB27A knockdown reduced this secretion and reversed the tumor-suppressive phenotypes (Wang X. et al., 2025). Thus, the tested knockout background supports a risk of secretion blockade, rather than a therapeutic rationale for inhibiting RAB27A across triple-negative breast cancer. It also illustrates why disease subtype and perturbation background must be retained when classifying candidate targets.

### 6.3 Exploiting autophagy-EV coupling to improve treatment response

Exploiting coupling includes both removal of immunosuppressive EV cargo and use of beneficial intercellular transfer. Temsirolimus, an mTOR inhibitor with established clinical use, promoted autophagy-dependent MVB degradation and reduced small-EV-associated programmed death ligand 1 (PD-L1) in preclinical tumor models; these changes accompanied improved T-cell responses and anti-PD-L1 activity (Park et al., 2022). This mechanism overlaps with lysosomal redirection in Section 6.1. It does not establish a clinically validated EV-specific indication, and systemic mTOR effects must be distinguished from the contribution of EV-associated PD-L1.

Beneficial extracellular transfer follows a different logic. The HMGB1 example in Section 5.3 illustrates how preserving a treatment-induced secretory response may improve chemosensitivity (Li et al., 2022). In hepatocellular carcinoma models, icaritin reduced expression of the glycolytic enzyme aldolase B (ALDOB) and transcription of the SNARE protein syntaxin 16 (STX16), impaired autophagolysosome formation, and promoted EV-associated transfer of autophagosomal material to macrophages. Degradation of the transcription factor signal transducer and activator of transcription 3 (STAT3) in recipient macrophages, together with altered macrophage survival or polarization, accompanied tumor suppression (Zheng et al., 2026). STX16 downregulation is a mechanistic component of this response, not evidence that icaritin directly binds STX16. These examples support context-specific use of endogenous cargo transfer; they do not establish engineered EV drug delivery or general safety of increasing secretion.

The principal opportunity is to influence treatment response through a defined donor–cargo–recipient relationship. The corresponding risk is that the same intervention changes other extracellular cargoes or exposes unintended recipient populations. Development should establish the contribution of the transferred cargo by depletion and rescue, assess tissue distribution and recipient exposure, and measure functional effects alongside donor-cell toxicity. Target engagement and a route-informed pharmacodynamic biomarker should precede claims of mechanism-based efficacy.

### 6.4 Comparative druggability and translational priorities

For druggability, the most tractable starting points in this set are agents with defined proximal pharmacology: VPS34 inhibition provides a kinase-directed tool with local xenograft combination evidence, whereas mTOR inhibition provides clinically used agents with preclinical EV-related functional evidence (New et al., 2017; Park et al., 2022). Neither is selective for the secretory interface. C1 offers *in vivo* pathway-redirection evidence but requires stronger linkage between exposure, TFEB dependence and the affected cargo route; asteltoxin remains an EV-modulating tool whose metabolic action complicates selectivity (Jiang et al., 2023; Mitani et al., 2022). TRIM16 and other trafficking candidates may identify narrower cell or cargo dependencies, but genetic efficacy must be translated into a selective pharmacological modality. These comparisons prioritize tool availability, target directness, functional evidence and selectivity separately, rather than assigning one universal target ranking.

## 7 Conceptual, methodological, and translational challenges

### 7.1 Defining routes and causal evidence

A central conceptual problem is that related extracellular outputs are often grouped under the same label. Direct secretory autophagy, LC3-positive EV secretion, lysosomal exocytosis, and lysosomal failure-driven extracellular disposal overlap, yet they are not equivalent (Buratta et al., 2020; Leidal and Debnath, 2021; Zubkova et al., 2024; Solvik et al., 2022). Lysosomal exocytosis is a distinct Ca2+-regulated surface-fusion route. TFEB overexpression increased lysosomal exocytosis through mucolipin 1 (MCOLN1), a lysosomal cation channel, and associated Ca2+ release (Medina et al., 2011). This result supports a clearance mechanism with its own terminal compartment, not a common final stage of secretory autophagy.

Recent work on autophagic extracellular vesicles suggests that at least some small EVs released under autophagy-inducing conditions are amphisome-derived and molecularly distinct from conventional exosomes (Mao et al., 2025). These vesicles are enriched in LC3B-II, p62, NBR1, Rab13, and selected ESCRT-III components, and depend on ATG7, Rab11, and Rab27a. These criteria are useful operationally, but they are best treated as an emerging classification rather than a settled taxonomy. Precision in route definition remains essential if the field is to mature (Noh et al., 2022; Pedrioli and Paganetti, 2021).

A useful next step is to test competing route assignments within the same model. A reduction in extracellular cargo after ATG disruption should be accompanied by measurements of intracellular cargo, lysosomal processing and cell integrity. Protease protection with and without detergent can distinguish lumenal cargo from externally associated material, as illustrated by SALI (Solvik et al., 2022). Genetic rescue and perturbations at different stages can then test whether loading and release are separable, as seen for ATG2/ATG9-dependent cargo loading and RAB11-dependent viral RNA packaging (Singh et al., 2026; Zhang et al., 2022). We propose this combination as a validation strategy, rather than treating any one marker as sufficient proof of origin.

### 7.2 Dynamic and *in vivo* route identification

Route assignment remains methodologically difficult *in vivo*. Detection of LC3 in extracellular vesicle fractions is not sufficient proof of one route over another, because LC3-positive extracellular material can arise in multiple contexts (Xu et al., 2022; Solvik et al., 2022; Mao et al., 2025). Live-cell assays for CD63-positive multivesicular body fusion, chemical screens, and older ultrastructural work help define candidate intermediates (Fader et al., 2008; Verweij et al., 2018; Cas et al., 2019). On their own, however, these approaches cannot distinguish direct secretory autophagy from EV-associated release. The amphisome is conceptually central yet technically difficult to capture as a secretion intermediate in intact tissues. Studies showing amphisome accumulation after HOPS loss underscore both its reality and the difficulty of inferring terminal fate from static images alone (van der Beek et al., 2024; Ganesan and Cai, 2021). Models such as amphiectosome-mediated release are likewise better viewed as proposed mechanisms than as general solutions to the field’s route-identification problem (Visnovitz et al., 2025). The route-assignment caveats in Table 1 and the contrasting model outcomes discussed in Section 3.3 illustrate why compartment accumulation or impaired fusion alone cannot establish increased secretion.

Dynamic imaging can address when cargo enters a secretory compartment and whether that compartment reaches the plasma membrane. A stabilized pH-sensitive CD63 reporter, combined with a non-pH-sensitive red tag, visualized MVB trafficking, fusion, EV uptake and endosomal acidification in cell models, with secretion also examined in three-dimensional culture and *in vivo* (Sung et al., 2020). Dual-color CD63 imaging has additionally resolved mechanically stimulated MVB–plasma membrane fusion events (Ji et al., 2025). These tools report CD63-associated trafficking, not secretory autophagy specifically. Combining them with cargo labeling, autophagy markers and route-selective perturbations could test whether a tracked carrier is an endosome or an amphisome; static colocalization alone would remain insufficient.

Single-EV measurements address a complementary question: whether a bulk change reflects altered vesicle abundance or a shift in cargo-positive subpopulations. Rolling circle amplification–expansion microscopy combines membrane-protein and microRNA measurements on individual EVs (Wu et al., 2025). Applied to defined donor-cell perturbations, such profiling could quantify cargo-positive fractions alongside total EV output and recipient-cell responses. However, post-isolation phenotyping cannot retrospectively establish membrane origin. Dynamic intracellular measurements and extracellular single-particle analysis should therefore be connected experimentally, with controls for labeling, co-isolation and cargo topology, rather than interpreted as interchangeable evidence (Solvik et al., 2022; Sung et al., 2020; Wu et al., 2025).

### 7.3 Cargo selection and pharmacological validation

Cargo identity currently provides some of the most useful route-based signatures, yet cargo selection remains incompletely understood. LDELS offers a well-defined example, in which LC3 conjugation machinery and LIR-related logic support export of RNA-binding proteins and TFRC (Leidal et al., 2020; Gardner et al., 2023; Leidal and Debnath, 2020). A distinct HSC70-LAMP2A pathway loads KFERQ-like proteins into a subset of exosomes, indicating that EV cargo selection can converge with autophagy-linked chaperone logic without being identical to LDELS (Ferreira et al., 2022). Many direct secretory autophagy cargoes, by contrast, including IL-1β and HMGB1, do not fit a simple LIR-based framework and appear to rely on cargo receptors, chaperones, or compartment-specific trafficking events (Kimura et al., 2017; Zhang et al., 2020; Kim et al., 2021; Chiritoiu et al., 2019). Organelle-scale cargoes complicate the picture further, since damaged mitochondria can be released by both autophagy-dependent and autophagy-independent routes depending on context (Tan et al., 2022; Liang et al., 2023). Endosomal microautophagy adds yet another layer of ambiguity by delivering soluble cytosolic proteins and selective autophagy receptors into late endosomal vesicles (Sahu et al., 2011; Mejlvang et al., 2018). Table 2 summarizes cargo classes, supporting observations and limits on route assignment. It remains unclear how cells distinguish cargo destined for secretion from cargo destined for degradation when both initially occupy overlapping autophagy-related membrane space.

Cargo selection should be tested independently of vesicle number. Comparing cargo abundance per vesicle, the fraction of cargo-positive vesicles and the intracellular cargo pool would help distinguish defective loading from reduced secretion or reduced synthesis. The separation between increased CD63-enriched EV release and impaired autophagic cargo loading after ATG2/ATG9 loss provides a concrete rationale for these measurements (Singh et al., 2026). Receptor or cargo-motif mutants with rescue could then test specificity, while lysosomal and recipient-cell assays would determine whether the intervention improves disposal or merely relocates the burden. This is especially important where intracellular retention and extracellular inflammatory effects diverge, as in SOD1 secretion models (Hutchings et al., 2024).

For pharmacological translation, the immediate priority is to connect target engagement to a defined route and a disease-relevant consequence. We propose advancing candidates only after dose- and time-dependent effects on cargo routing can be separated from loss of viable donor cells, global lysosomal failure or broad suppression of EV release. Genetic phenocopy and rescue should accompany pharmacological tools when feasible; donor-cell exposure should also be distinguished from direct effects of residual drug on recipient cells. Comparison in disease-relevant and protective secretory contexts would test the therapeutic window, given the divergent outcomes discussed in Sections 5 and 6 (Solvik et al., 2022; Li et al., 2022; Hutchings et al., 2024). After a reproducible cargo-specific effect is established, the next steps are tissue exposure and tolerability studies, a measurable pharmacodynamic marker, and confirmation in a disease-relevant model before mechanism-focused clinical testing. The cited studies do not establish that this full sequence has been completed for a selective interface intervention. Table 5 identifies the current evidence and the next validation step for representative interventions.

## 8 Conclusions and future directions

Autophagy-related extracellular output comprises several routes whose shared machinery does not imply a common membrane origin or biological effect. Cargo topology, lysosomal competence and cell-specific trafficking requirements determine how an extracellular signal should be interpreted (Leidal et al., 2020; Solvik et al., 2022; Singh et al., 2026). The therapeutic choice is consequently conditional: restoring degradation may reduce harmful export, suppressing selected secretion may limit inflammation or dissemination, and preserving or exploiting EV-linked communication may support treatment responses (Li et al., 2022; Wei et al., 2024; Hutchings et al., 2024). None of these directions can be chosen from EV abundance alone. The next advance should connect dynamic compartment tracking, cargo-resolved extracellular measurements and orthogonal perturbations to donor- and recipient-cell outcomes. Such evidence would support more selective pharmacological development; the studies reviewed here do not yet establish clinical efficacy through a specifically validated secretory-autophagy–EV mechanism.

## Glossary

AcbA: acyl-CoA-binding protein in *Dictyostelium*;
AGO2: Argonaute 2
ALDOB: aldolase B
ALIX: apoptosis-linked gene 2-interacting protein X (also known as PDCD6IP)
AMPK: AMP-activated protein kinase
ATG: autophagy-related
BECN1: beclin 1
CFTR: cystic fibrosis transmembrane conductance regulator
ER: endoplasmic reticulum
ERGIC: ER–Golgi intermediate compartment
ESCRT: endosomal sorting complexes required for transport
EV: extracellular vesicle
EV71: enterovirus 71
EVP: extracellular vesicle and particle
FAN: factor associated with nSMase2 activity
FIP200: focal adhesion kinase family-interacting protein of 200 kDa
FKBP51: FK506-binding protein 51 (encoded by FKBP5)
GABARAP: GABA type A receptor-associated protein
GABARAPL1: GABA type A receptor-associated protein-like 1
GCN2: general control nonderepressible kinase 2
GRASP: Golgi reassembly stacking protein
HER2: human epidermal growth factor receptor 2
HIF1A: hypoxia-inducible factor 1α
HMGB1: high mobility group box 1
HNRNPK: heterogeneous nuclear ribonucleoprotein K
HOPS: homotypic fusion and vacuole protein sorting
HSC70: heat shock 70-kDa protein 8
HSP90AA1: heat shock protein 90 alpha family class A member 1
IL-1β: interleukin-1β
ILV: intraluminal vesicle
IRE1α: inositol-requiring enzyme 1α
KARS1: lysyl-tRNA synthetase 1
LAMP2: lysosome-associated membrane protein 2
LAMP2A: lysosome-associated membrane protein 2A
LC3: microtubule-associated protein 1 light chain 3
LDELS: LC3-dependent EV loading and secretion
LIR: LC3-interacting region
LRRK2: leucine-rich repeat kinase 2
MCOLN1: mucolipin 1
MCT4: monocarboxylate transporter 4
MiT/TFE: microphthalmia family of basic helix-loop-helix-leucine-zipper transcription factors
MMP: matrix metalloproteinase
mTORC1: mechanistic target of rapamycin complex 1
MVB: multivesicular body
NBR1: neighbor of BRCA1 gene
nSMase2: neutral sphingomyelinase 2
OPTN: optineurin
PARK7/DJ-1: redox-sensitive protein PARK7 also known as DJ-1
PD-L1: programmed death ligand 1
PDCD6IP: programmed cell death 6-interacting protein
PI4P: phosphatidylinositol 4-phosphate
PLEKHG5: pleckstrin homology domain-containing family member 5
PLEKHM1: pleckstrin homology domain-containing family M member 1
PTEN: phosphatase and tensin homolog
PTP1B: protein tyrosine phosphatase 1B
RASAL2: Ras protein activator like 2
Rubicon: RUN domain Beclin-1-interacting and cysteine-rich domain-containing protein
SAFB: scaffold-attachment factor B
SALI: secretory autophagy during lysosome inhibition
SCFD1: Sec1 family domain-containing protein 1
SKA2: spindle and kinetochore-associated complex subunit 2
SKP1: S-phase kinase-associated protein 1
SNAP: synaptosome-associated protein
SNARE: soluble N-ethylmaleimide-sensitive factor attachment protein receptor
SOD1: superoxide dismutase 1
SQSTM1/p62: sequestosome 1
STAT3: signal transducer and activator of transcription 3
STX: syntaxin
TFE3: transcription factor binding to IGHM enhancer 3
TFEB: transcription factor EB
TFRC: transferrin receptor
TMED10: transmembrane emp24 domain-containing protein 10
TRIM16: tripartite motif-containing protein 16
TRIM56: tripartite motif-containing protein 56
ULK1: UNC-51-like kinase 1
UVRAG: UV radiation resistance-associated protein
V-ATPase: vacuolar ATPase
VAMP: vesicle-associated membrane protein
VPS34: vacuolar protein sorting 34, the class III phosphatidylinositol 3-kinase
WIPI2: WD repeat domain phosphoinositide-interacting protein 2 (WIPI2d denotes isoform d)
